# The epidemiology of human schistosomiasis in Gauteng Province, South Africa, 2017–2022

**DOI:** 10.4102/jphia.v16i4.1390

**Published:** 2025-08-29

**Authors:** Nchucheko Makhubele, Nqobile Ngoma, Tebogo Matjokotja, Peter S. Nyasulu, Mzimasi Neti, Refilwe Mokgetle

**Affiliations:** 1 South Africa Field Epidemiology Training Programme (SAFETP), National Institute for Communicable Diseases (NICD), Johannesburg, South Africa; 2 School of Health Systems and Public Health, Faculty of Health Sciences, University of Pretoria, South Africa; 3 Gauteng Provincial Department of Health (GDOH), Gauteng, South Africa; 4 African Health Research Institute (AHRI), Mtubatuba, South Africa; 5 Institute for Global Health, University College London, London, United Kingdom; 6 Department of Family Medicine, School of Medicine, Faculty of Health Sciences, University of Pretoria, South Africa; 7 Division of Epidemiology and Biostatistics, Department of Global Health, Faculty of Medicine and Health Sciences, Stellenbosch University, Stellenbosch, South Africa; 8 Division of Epidemiology and Biostatistics, School of Public Health, Faculty of Health Sciences, University of the Witwatersrand, Johannesburg, South Africa

**Keywords:** schistosomiasis, Gauteng Province, South Africa, trends, incidence, prevalence, distribution

## Abstract

**Background:**

Schistosimiasis affects over 250 million people globally. It is considered a moderately endemic condition in South Africa, with 36 people per 100 000 infected annually between 2011 and 2018. Despite its ability to cause long-term complications, it remains under-studied in Gauteng Province, and its epidemiological patterns are poorly understood.

**Aim:**

To describe the prevalence and trends of human schistosomiasis from 2017 to 2022.

**Setting:**

Gauteng Province, South Africa.

**Methods:**

A descriptive cross-sectional study was conducted using all clinical and laboratory human schistosomiasis cases in Gauteng Province from 2017 to 2022. Descriptive statistics summarised cases. Annual trends, seasonal patterns, and geographic distribution were assessed. Yearly incidence rates and overall provincial prevalence were estimated per 100 000 population.

**Results:**

There were 2526 human schistosomiasis cases recorded. The median age was 28 years (interquartile range [IQR]: 19–42), and men aged 10–19 years were most affected (15%). Cases declined by 37 per year over the study period, with seasonal peaks in February–March and August–September. The highest incidence occurred in 2019 (3.83 cases per 100 000). City of Tshwane Metropolitan and Mogale City had region-specific prevalence of > 25 cases per 100 000; overall provincial prevalence was 15 cases per 100 000.

**Conclusion:**

Enhanced surveillance, strengthened reporting, targeted awareness and preventative measures in vulnerable communities are recommended to reduce transmission. Ongoing research is crucial to inform evidence-based interventions in Gauteng Province and South Africa.

**Contribution:**

The study identified key demographic patterns, geographic hotspots, and temporal trends of human schistosomiasis cases in Gauteng Province.

## Introduction

Schistosomiasis, also known as bilharzia, is a neglected tropical disease (NTD) caused by trematode parasites of the genus *Schistosoma*, transmitted by snails that serve as intermediate hosts.^1,2^ The World Health Organization (WHO) estimated that as of 2023, schistosomiasis affected approximately 240 million people worldwide and 85% of infections were in underdeveloped areas of sub-Saharan Africa.^3,4^ In South Africa, 4 million people are at risk of contracting schistosomiasis, mostly children living in underserved communities.^4,5,6^ Furthermore, a study conducted in South Africa reported an average of 36 people per 100 000 infected with schistosomiasis annually between 2011 and 2018.^6^ South Africa is considered moderately endemic for schistosomiasis, with its sub-tropical climate providing suitable habitats for certain snail species in certain regions.^7^ The disease has been reported in the provinces of Limpopo, Mpumalanga, parts of Gauteng, KwaZulu-Natal, and the Eastern Cape.^1,4^

Two forms of the disease commonly exist in humans: namely, intestinal schistosomiasis caused by *Schistosoma mansoni* and *Schistosoma japonicum*, and urogenital schistosomiasis caused by *Schistosoma haematobium*, which is more common.^1,8^ It is considered a disease of poverty,^9^ common in areas with inadequate water and sanitation, and is the second most fatal parasitic disease.^1,4^ Transmission occurs when infected snails release schistosomes into freshwater, which can penetrate human skin upon contact with water.^1,4^ Infected humans can further contaminate freshwater by shedding schistosome eggs in urine and faeces. Activities such as swimming, bathing, farming, fishing, river washing and other water-related activities increase one’s exposure to infection.^3,10^

The disease can lead to severe, irreversible and potentially fatal complications. Reinfection has been associated with intestinal morbidity and urogenital disorders such as bladder fibrosis, hydronephrosis and an increased risk of bladder cancer in *S. haematobium* infections.^4,11^ *S. mansoni* and *S. japonicum* infections can result in periportal fibrosis, portal hypertension and splenomegaly – all of which are considerably life-threatening.^4^ It has also been noted to increase susceptibility to human immunodeficiency virus (HIV) among women because of genital lesions in female genital schistosomiasis.^11,12^ In children, *Schistosoma* infection can contribute to malnutrition, anaemia, cognitive dysfunction and growth inhibition.^2,6^ This leads to disrupted school attendance, classroom attendance and long-term psychosocial effects in children.

Schistosomiasis is a category 2 Notifiable Medical Condition (NMC) in South Africa, requiring notification within seven days of diagnosis by healthcare professionals and diagnostic laboratories.^13^ Schistosomiasis is underreported because of its dependence on laboratory detection, which is reliant on patients actively seeking healthcare.^6^ This results in an underestimation of the burden of disease in the country.

Despite schistosomiasis being previously reported in Gauteng, its prevalence and trends in the province are not well understood. Studies have explored aspects of the disease nationally and in other parts of the country where it is prevalent. However, very few studies have information on the disease in Gauteng Province.^6,14^ Furthermore, no studies have focused solely on the province by exploring its epidemiological trends to provide necessary locally relevant public health recommendations.

The WHO strategy to prevent and control schistosomiasis includes preventative chemotherapy through mass drug administration to at-risk populations alongside efforts to provide safe water access, adequate sanitation, health education and snail control, with the overall aim of reducing transmission of the disease as well as morbidity resulting from the disease.^3,10,15^ Schistosomiasis has been eliminated successfully in countries such as Japan, Tunisia and some parts of the Caribbean through extensive public health efforts implementing the WHO strategy to prevent and control the disease.^16^ While other countries such as China and Egypt have made significant progress in controlling the disease and moving towards ultimate elimination, it remains a public health problem in South Africa.^6,10^ In order to identify populations at risk and to inform public health action in Gauteng Province, studies exploring the different aspects of the disease need to be conducted.

This study aimed to investigate the epidemiology of human schistosomiasis in Gauteng Province from 2017 to 2022 by describing the demographic characteristics of human schistosomiasis cases determining the trends in human schistosomiasis cases in the province, and estimating the provincial prevalence of schistosomiasis.

## Research methods and design

### Study design

A cross-sectional study was conducted using a descriptive analysis of all clinical and laboratory-confirmed schistosomiasis cases reported in Gauteng Province, South Africa, between 2017 and 2022.

### Study setting

Gauteng Province is one of the nine provinces in South Africa. It is considered the financial hub of the country and contains the administrative capital. It is the most populous province, with a population estimated at 16.1 million according to mid-2022 estimates. There are five districts in the province: namely, Johannesburg, Tshwane, Ekurhuleni, West Rand and Sedibeng, and 27 sub-districts. Although the province is largely urbanised, various water bodies and streams are distributed across the districts, starting in the south and running clockwise around the province. These freshwater sources, combined with seasonal rainfall and sub-tropical climate conditions, provide suitable conditions for intermediate snail hosts, contributing to the transmission of schistosomiasis.^7^ Economic activities such as urban agriculture, informal car washes and construction near water sources, as well as domestic activities such as stream laundry washing, water collecting and bathing may increase the risk of exposure.

### Study population

The study comprised all schistosomiasis cases that were clinically and/or laboratory notified in the province from 01 January 2017 to 31 December 2022. According to South Africa’s NMC surveillance system, a case is laboratory-confirmed when schistosome eggs are detected in a patient’s urine or faeces or on histopathology on biopsy samples; or ≥ four-times increase in titre of serological test over 2 weeks; or repeated positive antigen test, and a suspected case refers to a person with clinical features of an acute infection (fever, hepatosplenomegaly, urticaria, diarrhoea, etc.) or intermediate infection (haematuria, cervicitis, etc.) or late infection (hydronephrosis, portal hypertension, etc.), and history of exposure in an endemic area.

### Data source

All human schistosomiasis cases reported through the NMC surveillance system and captured in the Gauteng Province schistosomiasis database between 01 January 2017 and 31 December 2022 were included in the study.

### Data analysis

Descriptive statistics were used to summarise the data and the characteristics of human schistosomiasis cases. Counts (*n*) and percentages (%) were used to summarise categorical variables, and median and interquartile range (IQR) to summarise numerical variables. To determine the trends in human schistosomiasis cases, the annual number of cases and monthly number of cases by year were determined to assess seasonal patterns. The yearly incidence rate of human schistosomiasis cases in Gauteng Province was calculated as the number of new cases reported per year divided by the mid-year population of that year and represented per 100 000 population. To estimate region-specific prevalence, the total number of cases in the region during the time of the study was divided by the mid-year population in that region and represented per 100 000 population. This was carried out to ensure normalisation required for developing a choropleth map using ArcGIS. To estimate the overall prevalence of human schistosomiasis in the province, the proportion of cases over the total population using the 2022 mid-year population estimate was calculated and represented per 100 000 population. Results were shown in tables, graphs and maps.

### Ethical considerations

Ethical clearance to conduct this study was obtained from the University of Pretoria Faculty of Health Sciences Research Ethics Committee (Ref: 663/2023). Data were provided by the Gauteng Department of Health Public Health Directorate, with all identifiable information removed prior to analysis. The dataset was securely stored as a password-protected file using Microsoft® Excel 2016.

## Results

There were 2526 human schistosomiasis cases in Gauteng Province from 01 January 2017 to 31 December 2022. The median age of cases was 28 years (IQR: 19–42), and 22.0% (*n* = 556/2526) of the cases were in the 20–29 years age group. Most of the cases were male (59.5%; *n* = 1504/2526). The City of Tshwane Metropolitan had the most cases (42.8%; *n* = 1081/2526) in the province, followed by Ekurhuleni Metropolitan (26.3%; *n* = 663/2526). Public health facilities reported 77.5% (*n* = 1959/2526) of the cases. Regarding case classification, 73.7% (*n* = 1861/2526) were laboratory notifications. Diagnostic testing confirmed 96.0% (*n* = 2425/2526) of cases through laboratory methods. Of all cases, 14.0% (*n* = 354/2526) were treated as outpatients, and the case fatality ratio was 0.2% (*n* = 5/2526) (Table 1).

**TABLE 1:** Characteristics of human schistosomiasis cases in Gauteng Province, South Africa, 2017–2022 (*N* = 2526).

Characteristic	Median	IQR	*n*	%
**Age (years)**	28	19–42	-	-
**Age group (years)**				
0–9	-	-	62	2.50
10–19	-	-	461	18.30
20–29	-	-	556	22.00
30–39	-	-	349	13.80
40–49	-	-	226	9.00
50–59	-	-	142	5.70
60+	-	-	181	7.20
Unknown	-	-	549	21.70
**Sex**				
Male	-	-	1504	59.50
Female	-	-	1020	40.40
Unknown	-	-	2	0.10
**Region†**				
Ekurhuleni Metropolitan	-	-	663	26.30
City of Johannesburg Metropolitan	-	-	571	22.60
City of Tshwane Metropolitan	-	-	1081	42.80
Sedibeng				
Emfuleni	-	-	5	0.60
Lesedi	-	-	13	0.50
Midvaal	-	-	0	0.00
West Rand				
Merafong City	-	-	51	2.00
Mogale City	-	-	119	4.70
Rand West City	-	-	13	0.50
**Facility sector**				
Public	-	-	1959	77.50
Private	-	-	567	22.50
**Classification on NMC surveillance system**				
Laboratory notification	-	-	1861	73.70
Clinical notification	-	-	665	26.30
**Diagnostic method**				
Laboratory	-	-	2425	96.00
Clinical signs and symptoms only	-	-	6	0.24
Rapid test	-	-	3	0.12
Other/Unknown			75	3.64
**Admission status**				
Discharged	-	-	225	8.90
Inpatient	-	-	146	5.80
Outpatient	-	-	354	14.00
Unknown	-	-	1801	71.30
**Outcome status**				
Alive	-	-	722	28.60
Died	-	-	5	0.20
Unknown	-	-	1799	71.20

† , Appendix 1 Table 1-A1 provided with expanded regions for metropolitans.

**FIGURE 1:**
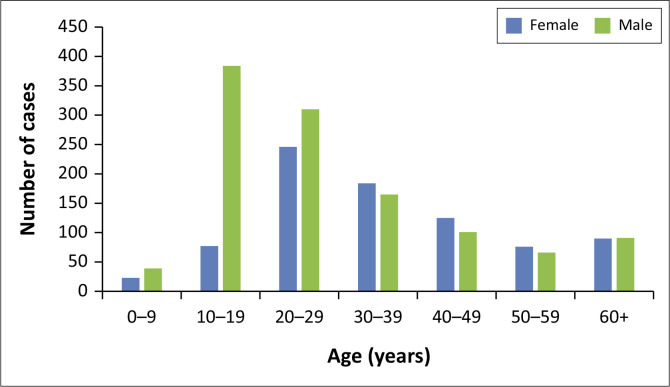
Distribution of human schistosomiasis cases by age and gender in Gauteng Province, South Africa, 2017–2022.

Men of the 10–19-year age group had the highest (384) number of cases, followed by men of the 20–29-year age group (310) (Figure 1).

**FIGURE 2:**
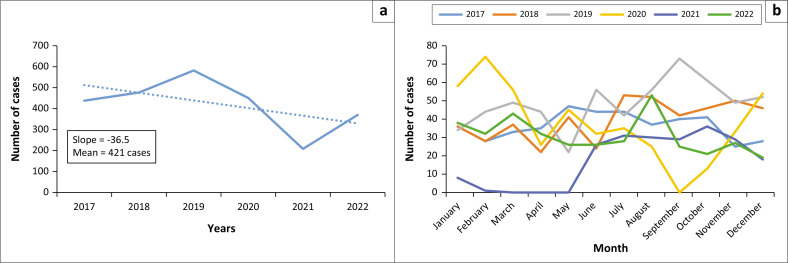
Distribution of human schistosomiasis cases by year (a) and by month (b) in Gauteng Province, South Africa, 2017–2022.

Gauteng Province experienced the highest number of human schistosomiasis cases (*n* = 582) in 2019 and the lowest (*n* = 208) in 2021. There was a mean of 421 cases per year and an overall declining trend of 37 cases per year between 2017 and 2022 (Figure 2).

The highest number of cases was reported in February 2020 (*n* = 74), followed by September 2019 (*n* = 73). Seasonal peaks in case numbers were observed in February–March and August–September across most years (Figure 2).

**FIGURE 3:**
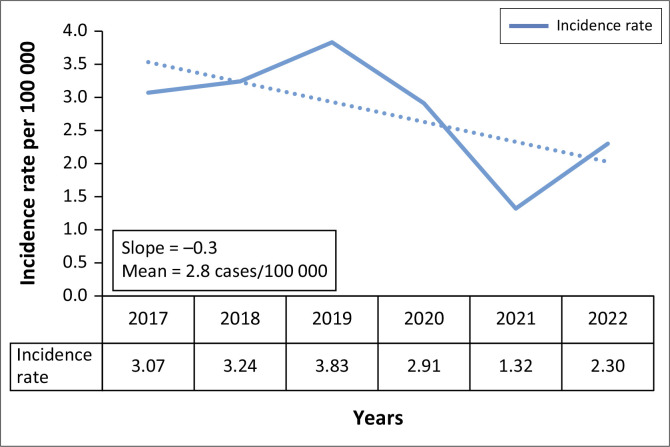
Yearly incidence rates of human schistosomiasis cases in Gauteng Province, South Africa, 2017–2022.

Gauteng Province experienced the highest human schistosomiasis incidence rate in 2019 (3.8 cases per 100 000 persons). The year with the lowest incidence rate was 2021 (1.3 cases per 100 000 persons). The average provincial incidence rate was 2.8 cases per 100 000 persons between 2017 and 2022 (Figure 3).

**FIGURE 4:**
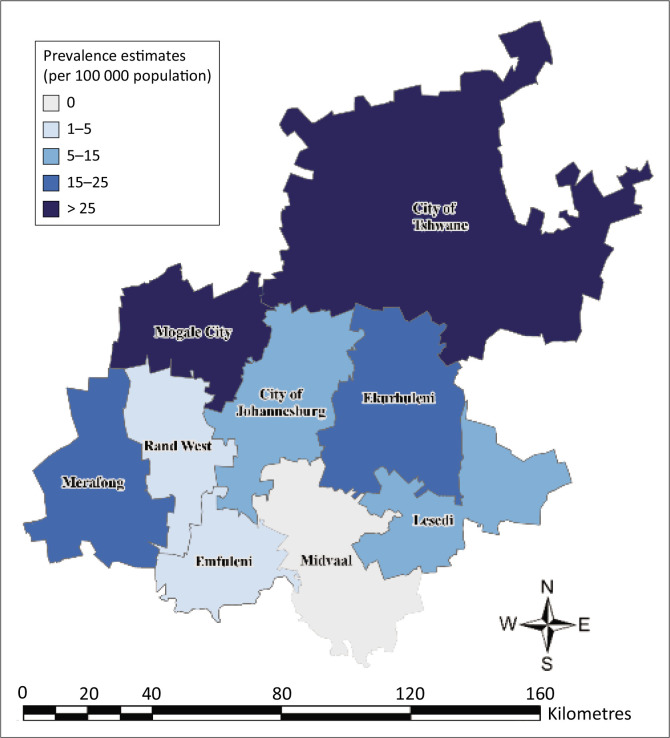
Region-specific prevalence estimates of human schistosomiasis in Gauteng Province, South Africa, 2017–2022.

The overall prevalence of human schistosomiasis in Gauteng Province was 15 cases per 100 000 between 2017 and 2022. The regions in the province with the highest human schistosomiasis prevalence estimate were Mogale City and City of Tshwane Metropolitan (27 cases per 100 000). The Midvaal region had no cases (Figure 4).

## Discussion

Schistosomiasis remains an NTD of public health concern in South Africa, particularly in disadvantaged communities with inadequate access to water and sanitation. This study aimed to describe the epidemiology of human schistosomiasis to assess trends, geographic distribution and demographic characteristics of reported cases, particularly in Gauteng Province, where the disease remains under-studied and thus not well understood.

In our study, we found that most of the human schistosomiasis cases were young adults, with a high proportion of cases among men aged 10–19 years. Most of the cases were from the public sector, were confirmed cases, and were treated as outpatients. There was a declining trend in the number of cases over time, as well as an overall decrease in incidence. Most of the cases were from the City of Tshwane Metropolitan, in the northern region of the province. The overall prevalence was 15 cases per 100 000.

Schistosomiasis is predominantly a disease affecting school-aged children, typically those defined as between 7 years and 15 years in South Africa.^4,6,17^ In this study, most cases were found in the young adult population aged 20–29 years, rather than school-going children. This could be the result of occupational and/or economic or recreational water-related activities, such as swimming, fishing, and informal work near water streams, in areas where schistosomiasis is more common. While previous literature has reported on children younger than 19 years being infected, reinfection has been a common occurrence even after successful treatment of the first episode.^18,19^ This may contribute to a similar prevalence of schistosomiasis between the two age groups (school-aged children and young adults) as seen in our study, suggesting a possible continued exposure to infested water sources, leading to repeated infections over time. Further research is necessary to understand reinfection patterns.

Furthermore, age- and sex-specific trends showed that men aged 10–19 years had more cases. This aligns well with the suggestion that school-going boys in endemic areas are more likely to engage in recreational water-related activities, thus increasing their risk of exposure.^4^ While literature has suggested that women are most affected by schistosomiasis because of their involvement in washing clothes and bathing children in open water sources in lower-income settings,^9,20^ our study found that prevalent cases were more in men across most age groups. This finding is similar to previous studies carried out in South Africa, which described a higher prevalence of schistosomiasis in men.^5,6,17,21^ Among women, the highest burden was observed in those aged 30–59 years, which is more likely the ages of women partaking in domestic labour involving water contact. These trends support the idea that the risk of exposure may vary by gender and age because of different water contact behaviours. Gender disparities and local differences in prevalence may also be influenced by the *Schistosoma* species, known to affect men and women differently.^20^ This highlights the need for exploring the type of species more prevalent in high-burden regions in Gauteng Province to inform targeted control measures.

The study also found that Gauteng Province experienced an overall declining trend in the number of human schistosomiasis cases between 2017 and 2022. This could be the result of the coronavirus disease 2019 (COVID-19) pandemic, as mobility and social activities were restricted, reducing movement during this period, thus reducing exposure to water-related activities. In addition, the pandemic led to widespread disruption in routine public health services and surveillance, which may have affected reporting and ultimately underestimated the incidence. The subsequent increase in cases from 2021 may reflect a resumption of healthcare services and routine surveillance as public health systems began to stabilise.

Monthly trends in our study revealed fluctuations in schistosomiasis cases, with peaks observed in February–March and August–September. These periods coincide with increased water-related activities because of seasonal factors. The early-year peak may be linked to summer rainfall, characteristic of the sub-tropical climate in Gauteng Province, creating favourable conditions for reproduction of snails. The late-year peak could correspond with post-winter warming, leading to an increase in human–water-related activities. This is consistent with evidence suggesting that schistosomiasis prevalence is higher during hot, rainy seasons.^7,12^ Lower cases in May–June (early winter) and November–December (early summer) may reflect reduced exposure as a result of colder temperatures or other environmental factors affecting the likelihood of transmission.

Furthermore, our study found that most of the cases were classified as laboratory notifications. This classification is significant within the NMC framework, as laboratory notifications indicate more definitive diagnoses, while clinical notifications represent suspected cases notified by clinicians before laboratory testing. Additionally, in terms of diagnostic methods, almost all cases were confirmed through laboratory testing such as microscopy or antigen detection. Although this is a requirement for schistosomiasis, this finding revealed a gap that exists between diagnostic testing and epidemiological classification. The observed proportion of clinical notifications suggests potential lapses in reporting, which may be a result of delays or a lack of updating case statuses once laboratory results become available. This highlights the need for strengthened surveillance through having complete and accurate reporting of data related to schistosomiasis case records.

City of Tshwane Metropolitan, north of the province, had the highest proportion of schistosomiasis cases in the province and a high region-specific prevalence per 100 000 population. This finding aligns with evidence that northern parts of Gauteng Province are endemic for schistosomiasis.^1^. Notably, Mogale City had a high region-specific prevalence (> 25 cases per 100 000) despite contributing relatively few cases to the provincial total. Unlike City of Tshwane and other metropolitan areas, Mogale City has a smaller population, suggesting a high disease burden, possibly because of various environmental, socio-economic, occupational, migration, and healthcare-related factors.

Our study had a few limitations. Firstly, the database used in the study did not include data on socio-economic factors, which could have provided a better insight into understanding drivers of schistosomiasis prevalence in the province. Secondly, the dataset had limited variables, restricting the ability to conduct a risk factor analysis that would provide a clear understanding of exposure-risk associations. Thirdly, a considerable proportion of cases had an unknown age, which may have affected age-specific analysis and interpretation. Despite these limitations, this is the first study to document the epidemiology of human schistosomiasis in Gauteng Province, South Africa.

## Conclusion

Schistosomiasis was found to be predominant among young adults and notably present among men of all ages. It was most prevalent in the City of Tshwane district, and there was a general downward trend of cases during this period of the study. We recommend that NTDs such as schistosomiasis be prioritised, with strengthened surveillance and reporting of human schistosomiasis in both public and private healthcare sectors for appropriate public health interventions to control the continued transmission of the disease, known to lead to chronic complications affecting the heart and liver, among others.

## Appendix 1

**TABLE 1-A1:** Distribution of human schistosomiasis cases by health sub-district in Gauteng Province, South Africa, 2017–2022 (*N* = 2526).

Characteristic	*n*	%
**Sub-district**	170	6.7
Ekurhuleni sub-district A	246	9.7
Ekurhuleni sub-district B	13	0.5
Ekurhuleni sub-district C	65	2.6
Ekurhuleni sub-district D	45	1.8
Ekurhuleni sub-district E	124	4.9
Ekurhuleni sub-district F	15	0.6
Emfuleni	13	0.5
Lesedi	51	2.0
Merafong City	119	4.7
Mogale City	13	0.5
Rand West City	36	1.4
Johannesburg A	128	5.1
Johannesburg B	17	0.7
Johannesburg C	171	6.8
Johannesburg D	58	2.3
Johannesburg E	137	5.4
Johannesburg F	24	1.0
Johannesburg G	187	7.4
Tshwane 1 sub-district	34	1.3
Tshwane 2 sub-district	642	25.4
Tshwane 3 sub-district	40	1.6
Tshwane 4 sub-district	33	1.3
Tshwane 5 sub-district	126	5.0
Tshwane 6 sub-district	13	0.5
Tshwane 7 sub-district	-	-
